# Annoyance and activity disturbance induced by high-speed railway and conventional railway noise: a contrastive case study

**DOI:** 10.1186/1476-069X-13-12

**Published:** 2014-03-07

**Authors:** Guo-Qing Di, Qi-Li Lin, Zheng-Guang Li, Jian Kang

**Affiliations:** 1 Institute of Environmental Pollution & Control Technology, Zhejiang University, Hangzhou 310058, China; 2 School of Architecture, University of Sheffield, Western Bank, Sheffield S10 2TN, UK

**Keywords:** Noise descriptor, High-speed railway, Evaluation index, Annoyance, Activity disturbance

## Abstract

**Background:**

High-speed railway (HR, Electrified railway with service speed above 200 km/h.) noise and conventional railway (CR, Electrified railway with service speed under 200 km/h.) noise are different in both time and frequency domain. There is an urgent need to study the influence of HR noise and consequently, develop appropriate noise evaluation index and limits for the total railway noise including HR and CR noise.

**Methods:**

Based on binaural recording of HR and CR noises in a approximate semi-free field, noise annoyance and activity disturbance induced by maximal train pass-by events in China were investigated through laboratory subjective evaluation. 80 students within recruited 102 students, 40 males and 40 females, 23.9 ± 2.1 years old, were finally selected as the subjects. After receiving noise stimulus via headphone of a binaural audio playback system, subjects were asked to express the annoyance or activity disturbance due to railway noise at a 0-100 numerical scale.

**Results:**

The results show that with the same annoyance rating (*A*) or activity disturbance rating (*D*), the A-weighted equivalent sound pressure level (*L*_Aeq_) of CR noise is approximately 7 dB higher than that of HR noise. Linear regression analysis between some acoustical parameters and *A* (or *D*) suggests that the coefficient of determination (R^2^) is higher with the instantaneous fast A-weighted sound pressure level (*L*_AFmax_) than that with *L*_Aeq_. A combined acoustical parameter, *L*_HC_ = 1.74*L*_AFmax_ + 0.008*L*_AFmax_(*L*_p_-*L*_Aeq_), where *L*_p_ is the sound pressure level, was derived consequently, which could better evaluate the total railway noise, including HR and CR noise. More importantly, with a given *L*_HC_, the noise annoyance of HR and CR noise is the same.

**Conclusions:**

Among various acoustical parameters including *L*_HC_ and *L*_Aeq_, *A* and *D* have the highest correlation with *L*_HC_. *L*_HC_ has been proved to be an appropriate index to evaluate the total railway noise, including both HR and CR. However, it should be pointed out that this study provides suggestive evidence, rather than a final proof. Further study is expected to elucidate conclusions above by additional measurements.

## Background

The research on high-speed railway (HR) noise has attracted more and more attention. The train noise consists of various sources, such as wheel/rail noise and aerodynamic noise. The aerodynamic noise depends strongly on train speed [1]. In a study of Shinkansen train, which is the high-speed railway train in Japan with the maximum speed of 240 km/h ~320 km/h, it was shown that the aerodynamic noise was dominant sound source of the train noise when the train speed exceeded 270 km/h [2,3]. Moreover, the duration of HR noise is normally shorter than that of conventional train noise. Consequently, the HR noise and conventional railway (CR) noise are different in both time and frequency domain. Compared with CR noise, the magnetic levitation railway (MLR) noise is more similar to HR noise [4,5].

The results obtained by field surveys or laboratory studies could provide supports for setting standards for traffic noise. A number of field surveys on CR noise have been carried out in different countries [6]. Commercial HR systems have been built in a number of countries, including Japan, France and China, but there have been limited reports on the field surveys of HR noise. When Shinkansen was opened in 1964, its noise caused a lot of complaints. As there was no special evaluation standard for this new traffic noise, a field survey was carried out by the Environmental Agency of Japan. Based on the results, the maximum A-weighted equivalent sound level, *L*_ASmax_, with the time duration as 1000 ms, was selected to evaluate the HR noise [7]. In 1993, a field survey was carried out to assess the impact of noise induced by the TGV Atlantique line in France [8]. Whilst the A-weighted equivalent sound pressure level, *L*_Aeq_, seemed to be a relevant noise annoyance descriptor for the daytime, the number of noise events or the length of time over 70 dB (A) seemed to be more appropriate for the evening. Based on a field survey in Shanghai on the magnetic levitation rail (MLR) noise impact, Chen et al [9] found that *L*_ASmax_ is more appropriate than *L*_Aeq_ as a noise descriptor.

Meanwhile, some researchers have compared the impact of the noises from CR, HR and MLR under laboratory conditions. In two relevant laboratory studies reported by Fastl and Gottschling [10,11], the overall (or global) loudness ratings for the MLR and CR noise, with the same *L*_Aeq_, were not significantly different. However, in a laboratory study reported by Neugebauer and Ortscheid [12], the subjects were more negative about MLR than CR. Moreover, especially at a higher *L*_Aeq_, the sounds from MLR were considerably louder than those of CR. In a laboratory study reported by Vos [13], the annoyance degree caused by MLR was also considerably higher than that by CR. In a study reported by De Coensel et al [5], a holiday cottage was selected as a natural setting, and subjects were asked to engage in simple daily activities during the tests. Using fixed outdoor loudspeakers, railway noise was reproduced. After noise exposure, subjects were asked to evaluate the noise annoyance. The results, however, showed that there was no significant difference among the annoyances caused by CR, HR or MLR noise at same *L*_Aeq_. Overall, the above results are inconsistent, which might be caused by the use of different stimuli. According to a laboratory study reported by Di et al [14], there is a strong correlation between the annoyance and the burst duration and interval time of intermittent noise. In the studies reported by Fastl and Gottschling [10,11], Neugebauer and Ortscheid [12] and Vos [13], pass-by sounds were used and each stimulus included only one noise event. The stimuli used by De Coensel et al [5] included several pass-by sounds.

In China, the total mileage of HR has exceeded 6900 km since the first HR was put into operation in 2008. However, no special sound environment quality standard for the area along HR has been set up in China. GB 3096-2008 [15], which is an “Environmental Quality Standard for Noise” of China, provides evaluation indices and noise limits for different sound environment function areas including the area along CR, i.e. the existing noise limit developed for CR noise is also used for HR. Many residents living along the HR have complained about the noise impact from HR [16,17], so that there is an urgent need to study the influence of HR noise and consequently, develop appropriate noise index and limits.

In this study, therefore, based on binaural recording of HR and CR noises in a approximate semi-free field, the noise annoyance and activity disturbance have been investigated through laboratory subjective evaluation. The aim is to examine the effects of different acoustical parameters, develop appropriate noise evaluation methods and indicators of total railway considering the two kinds of railway noise.

## Methods

### Binaural recording

The Artificial Head Measurement System HMS IV.0 (Head Acoustics, 2008) was used to sample HR and CR noises, and a digital video recorder was used to record the pass-by trains. The ear height was 1.2 m above the ground [15].

Acoustical characteristics of railway noise are pertaining to train type and rail track. The most representative train type and rail track was chosen in this study in order to that the research results can be generalized to railway system of China as far as possible. It was also confirmed by the Ministry of Railways of the People’s Republic of China that the train type and rail track of HR and CR chosen below has been most widely used now and will be generalized in the future in the railway network of China, especially in the high-speed railway network.

The HR noise was recorded in a semi-free open field along the Shanghai-Hangzhou HR, where rail level was 15 m higher than ground and the background noise was lower than 35 dBA. The recording was made at different distances from the HR, reflecting the attenuation characteristics. In an experiment reported by Kurra [18], the noise was recorded at 30 m and 100 m from the outboard track. In the experiment reported by Vos [13], the noise was recorded at 25 m, 50 m and 100 m from the outboard track. In the experiment reported by De Coensel et al [5], the recording distance was 25 m, 50 m, 100 m and 200 m from the outboard track. In this study, the recording distance was 20 m, 30 m, 50 m, 100 m and 200 m from the outboard track. During the recording, the pass-by trains were of same type (CRH380A, 8 railroadcars), and the speed was 301 ± 3.6 km/h.

The CR noise was recorded in a semi-free open field along the Zhejiang-Jiangxi CR, where rail level was 2.3 m higher than ground and the background noise was also lower than 35 dBA. The recording distances corresponded to those of HR recording. During the recording, the pass-by trains were of same type (SS8, 17 railroadcars), and the speed was 107 ± 10.7 km/h.

Considering that train noise actually varies from train to train even if the trains are moving with the same speed as the wheel conditions for different trains are not identical, the pass-by trains of same type were recorded 8 times at each distance from the outboard track.

### Test stimuli

**Figure 1:**
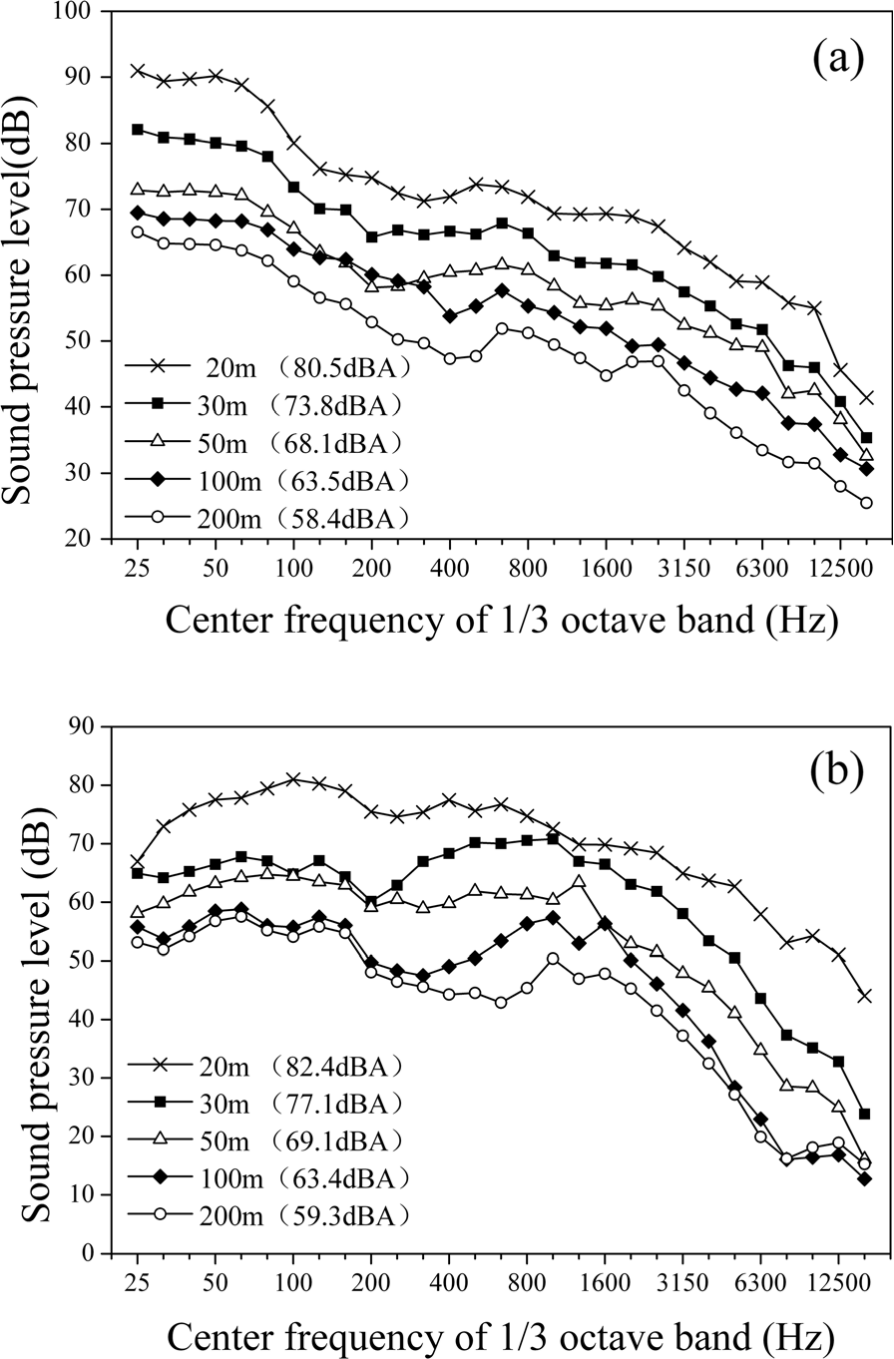
**SPL in 1/3**-**octave bands for 10 sound fragments. (a)** High-speed train (HR); **(b)** Conventional train (CR).

**Figure 2:**
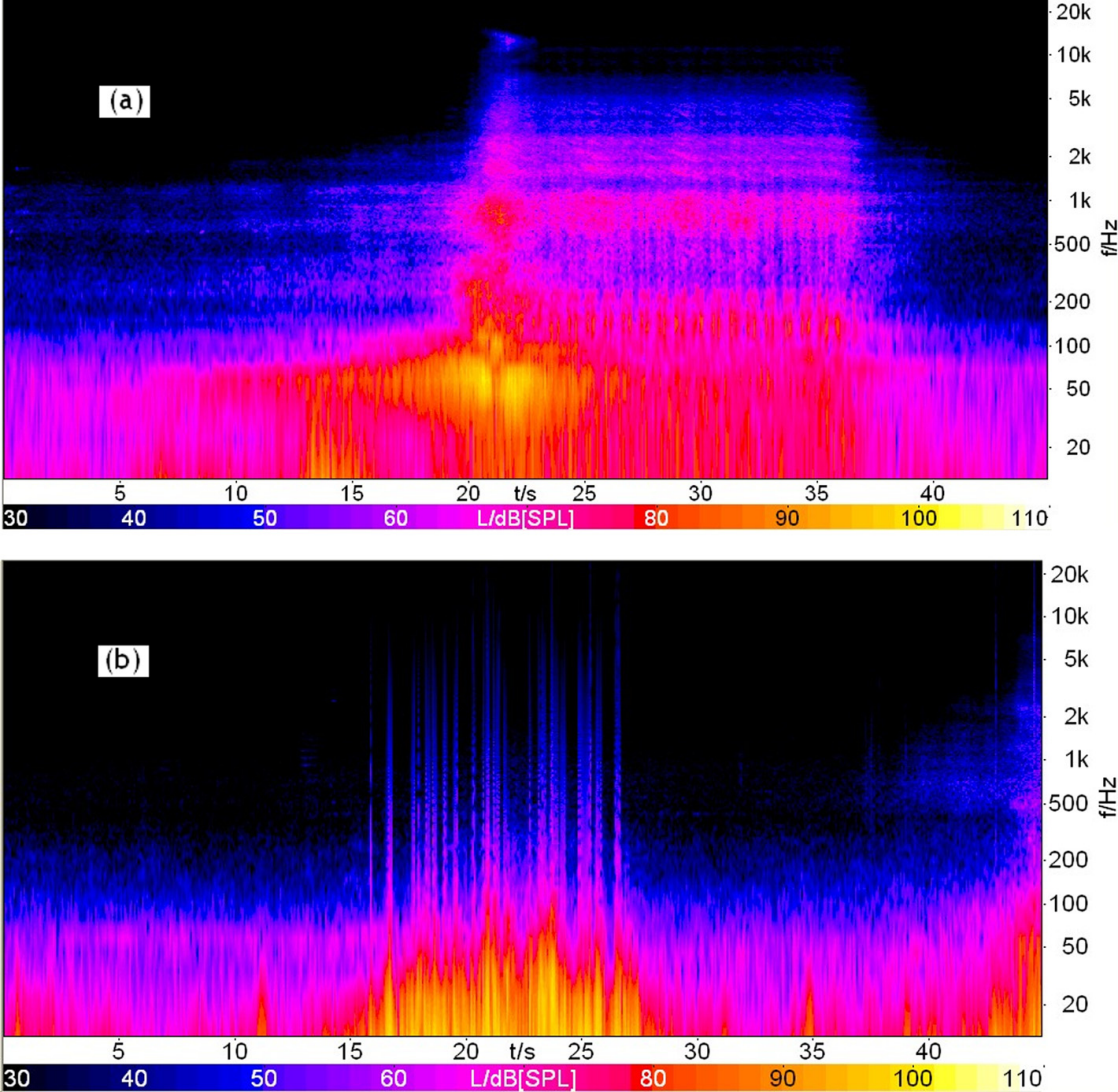
**A time-frequency analysis of railway noises recorded at 20 m from the outboard track. (a)** CR; **(b)** HR.

The recordings were analyzed using the ArtemiS 10.0 software (Head Acoustics, 2008). It was shown that the duration of pass-by sound was 15-20 s for each HR, and 35-40 s for each CR. In order to include the whole pass-by sound, the total duration of each sound fragment was fixed as 45 s [5,13], with the maximum level at about 22.5 s. The standard errors of *L*_ASmax_ and *L*_Aeq_ over 45 s for 8 recorded pass-by trains at the same distance from the outboard track were 3.0 dB and 2.0 dB, respectively. One recorded pass-by train, whose *L*_ASmax_ and *L*_Aeq_ is closest to the average, was then chosen among 8 recorded pass-by trains. Finally, ten sound fragments were prepared, five for HR and five for CR, each corresponding to a distance from the outboard track. The total level of the noise sample *L*_S_ (dB) is calculated [19] by

(1) $$L_{S}=\mathrm{lg}\frac{10^{L_{L}}+10^{L_{R}}}{2}$$

where *L*_L_ is the level of the left signal (dB), and *L*_R_ is the level of the right signal (dB). The difference between *L*_L_ and *L*_R_ is less than 1.0 dB.The sound pressure level (SPL) in 1/3-octave bands are illustrated in Figure 1 for each sound fragment. It can be seen that with increasing frequency, the HR SPL decreases continuously, and this tendency is relatively less significant for CR. To examine this in more detail, a time-frequency analysis of the noise recorded at 20 m from the outboard track has been carried out, as shown in Figure 2. It can be seen that the low frequency noise of HR is more prominent compared with CR.

In the laboratory study of traffic noise reported by De Coensel et al [5], Kurra [18], Vogt [20] and Sandrock et al [21], the test duration for subjective evaluation was 10-30 min. In the experiments where the duration of 30 min was used, subjects were asked to engage in two activities [18]. In other experiments, subjects were asked to engage in only one activity. In this study, 15 min was used as the test duration, which included six pass-by sounds with a constant interval that was determined based on train schedule, where the maximum train pass-by was approximately 24 per hour. In the studies carried out by De Coensel et al [5] and Kurra [18], a similar train frequency was used. During the test of activity disturbance, subjects were asked to engage in one activity, as described below.

### Subjective evaluation

The noise annoyance and activity disturbance induced by maximal train pass-by events in China (six pass-by sounds with a constant interval over 15 min) were investigated through laboratory subjective evaluation.

The binaural audio playback system consisted of four headphones (Sennheiser HD-600), a distribution amplifier (Head AcoustiAS HDA IV. 1) and a digital equalizer (Head AcoustiAS PEQ V). Headphones outputs were calibrated at the calibration laboratory of the HEAD acoustics GmbH. The differences of sound pressure level between nominal value and actual value for left channel or right channel of Headphones are all lower than 0.05 dB. The experiment was performed in a soundproof room (4 m by 4 m by 3 m), where the background noise was lower than 25 dBA.

In the first stage 102 students were recruited to take part in a hearing test, which was performed using an audiometer to test the hearing loss of each student. After excluding one student having an obvious hearing loss (>15 dB) [22] and 21 students whose curriculum schedules were conflicting with scheming experimental timetable, 80 students, 40 males and 40 females, 23.9 ± 2.1 years old, were finally selected as the subjects for subjective evaluation experiments. Since the binaural audio playback system consisted of four sets of headphones, only four subjects could receive noise exposure at the same time through headphones. 80 subjects were divided into 20 groups randomly. Each group included 4 subjects. Two independent experiments, annoyance and activity disturbance induced by railway noise, were carried out, respectively [23].

A numeric rating scale [24,25] was used to evaluate annoyance and activity disturbance induced by noise in this study. Before experiments, the subjects were told that the purpose of this experiment was to evaluate the annoyance and activity disturbance induced by railway noise. They were also asked to imagine they were in the outdoor environment and the recorded environmental background noise and video along the railway were presented by a dodecahedron loudspeaker and a television [26]. When experiments began, the dodecahedron loudspeaker was turned off and subjects received noise stimulus via headphone of a binaural audio playback system above. Previous study [27,28] reported that the visual settings influenced judgments on annoyance induced by noise. In order to reduce the impact of visual setting on the study, it is necessary to reproduce the original scene truly. In this study, the recorded video, including pass-by trains, was played back simultaneously with sounds. In the experiment of activity disturbance induced by noise, subjects were asked to select magazines to read individually during noise exposure [26]. After above noise exposure, subjects were asked to express the annoyance or activity disturbance due to railway noise at a 0-100 numerical scale [29], where 0 means ‘Not at all’, and 100 means ‘Extremely’ [24,30-33]. Since all the subjects in this study were university students with good English level, the evaluation sheet was both in English and Chinese.

### Data processing and statistical analysis

The annoyance rating (*A*) and activity disturbance rating (*D*) of each noise sample was calculated by averaging scores chosen by all subjects. Acoustical parameters (such as *L*_Aeq_, *L*_AFmax_, *L*_N_, etc.) of each noise sample were calculated by ArtemiS 10.0 software. All statistical analyses were performed by Origin 7.5 (OriginLab Corporation, Northampton, MA, USA). Three kinds of regression models, including linear model, second-order polynomial model and exponential model, were used to examine relationships between *A* ( *or D*) and each single acoustical parameter above. The regression model with the highest determination coefficient was given. In order to further develop an appropriate evaluation index for the total railway noise including HR and CR, a multiple regression model was used to examine relationships between combined acoustical parameters and *A* ( *or D*) of overall railway noise, including HR and CR. *L*_AFmax_ and *L*_p_-*L*_Aeq_ were chosen as acoustical parameters in multiple regression model which was given as below.

(2) $$A=a_{1}L_{\mathrm{AFmax}}+b_{1}L_{\mathrm{AFmax}}\left(L_{p}-L_{\mathrm{Aeq}}\right)–c_{1}$$

(3) $$D=a_{2}L_{\mathrm{AFmax}}+b_{2}L_{\mathrm{AFmax}}\left(L_{p}-L_{\mathrm{Aeq}}\right)-c_{2}$$

where a_1_, a_2_, b_1_, b_2_, c_1_, c_2_ are all constant terms.

## Results

**Figure 3:**
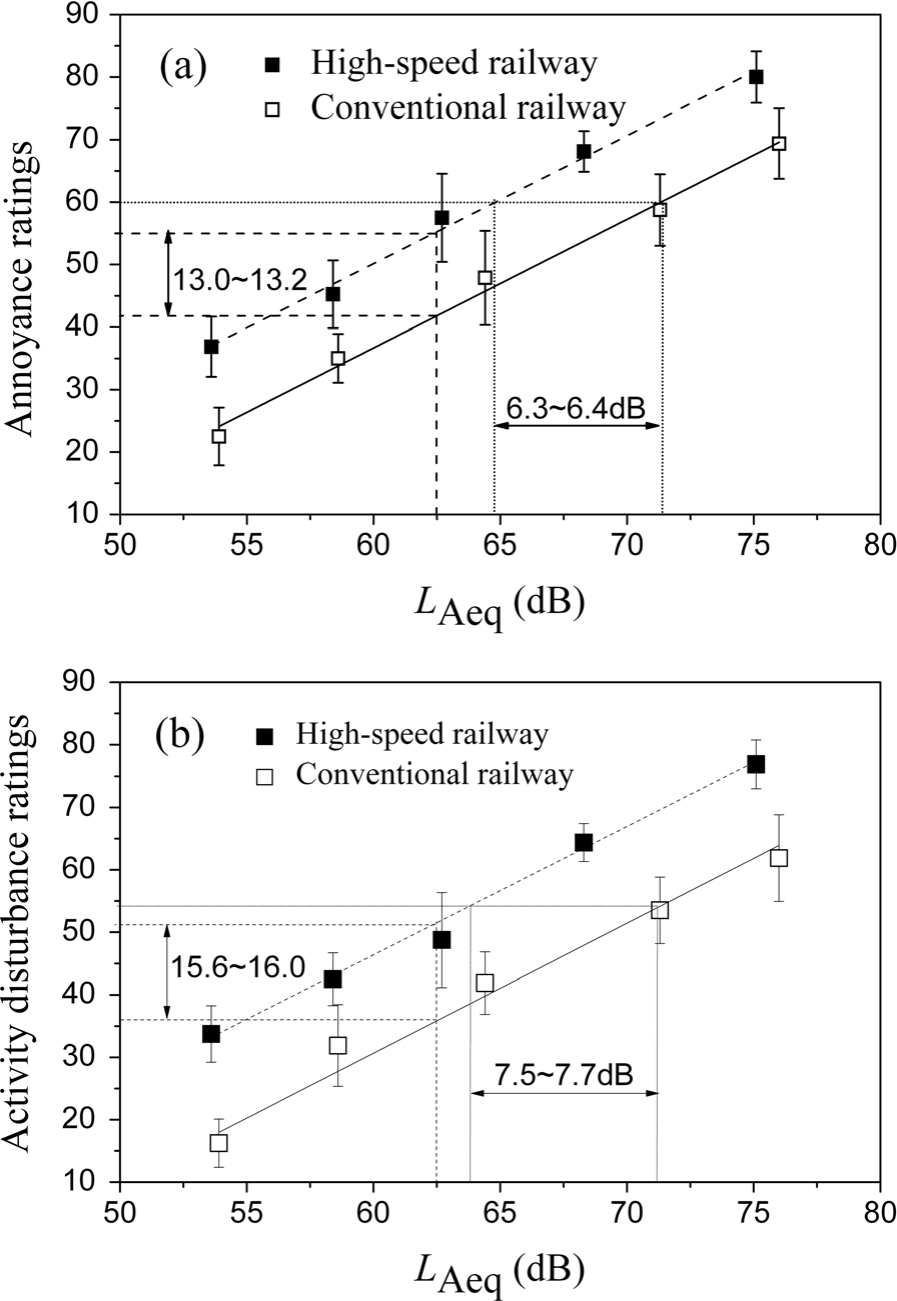
**The relationships between *A *and *L***_**Aeq**_**, and between *D *and *L***_**Aeq**_**. (a)** Between *A* and *L*_Aeq_. HR: *A* = 2.05*L*_Aeq_-72.9 (*R*^2^ = 0.993); CR: *A* = 2.06*L*_Aeq_-86.6 (*R*^2^ = 0.991); **(b)** Between *D* and *L*_Aeq_. HR: *D* = 2.06*L*_Aeq_-77.0 (*R*^2^ = 0.994); CR: *D* = 2.08*L*_Aeq_-94.1 (*R*^2^ = 0.978).

The relationships between the subjective evaluation results and *L*_Aeq_ of stimuli were then analyzed. Figure 3 (a) shows the relationship between *A* and *L*_Aeq_. It can be seen that *A* increases with increasing *L*_Aeq_. Based on comparing the coefficient of determination (*R*^2^) of three kinds of regression models (linear model, second-order polynomial model and exponential model), linear regression model having highest *R*^2^ was chosen to describe the relationship between *A* and *L*_Aeq_. The model for HR is given below, where the coefficient of determination (*R*^2^) is 0.993,

(4) $$A=2.05L_{\mathrm{Aeq}}-72.9$$

For CR, the model is given below, where *R*^2^ = 0.991,

(5) $$A=2.06L_{\mathrm{Aeq}}-86.6$$

From the above it can be derived that with the same *L*_Aeq_, *A* of HR noise is approximately 13.0-13.2 higher than that of CR noise. With the same *A*, *L*_Aeq_ of CR noise is approximately 6.3-6.4 dB higher than that of HR noise. Compared with CR noise, for HR noise the peak level of train events (*L*_Amax_) and the proportion of low frequency sound energy are generally higher and the duration of noise is shorter [8,10,14], which could result in greater annoyance of HR noise compared with that of CR noise at the same *L*_Aeq_.

Figure 3 (b) shows the relationship between *D* and *L*_Aeq_. Similar to the relationship between *A* and *L*_Aeq_, with increasing *L*_Aeq_, *D* increases, and the relationship for HR can be given below, where the coefficient of determination (*R*^2^) is 0.994,.

(6) $$D=2.06L_{\mathrm{Aeq}}-77.0$$

For CR, the relationship between *D* and *L*_Aeq_ is given below, where *R*^2^ = 0.994,

(7) $$D=2.08L_{\mathrm{Aeq}}-94.1$$

It can be derived that with the same *L*_Aeq_, *D* of HR noise is approximately 15.6-16.0 higher than that of CR noise, whereas with the same *D*, *L*_Aeq_ of CR noise is approximately 7.5-7.7 dB higher than that of HR noise.

Using multiple regression model, the relationships between combined acoustical parameters and *A*, *D* of overall railway noise, including HR and CR, were obtained as Eq. (8) and Eq. (9), where *R*^2^ are 0.991 and 0.977, respectively.

(8) $$A=1.74L_{\mathrm{AFmax}}+0.008L_{\mathrm{AFmax}}\left(L_{p}-L_{\mathrm{Aeq}}\right)-89.7$$

(9) $$D=1.74L_{\mathrm{AFmax}}+0.008L_{\mathrm{AFmax}}\left(L_{p}-L_{\mathrm{Aeq}}\right)-94.8$$

Defining *L*_HC_ = 1.74*L*_AFmax_ + 0.008*L*_AFmax_(*L*_p_-*L*_Aeq_), equations above can be simplified to Eq. (10) and Eq. (11) as below.

(10) $$A=L_{\mathrm{HC}}-89.7$$

(11) $$D=L_{\mathrm{HC}}-94.8$$

Comparing with linear regression models having single acoustical parameter in Table 1, the determination coefficient (*R*^2^) of the multiple regression model having combined acoustical parameters is higher.

**Table 1:** **The relationships between the subjective evaluation results** (**both HR and CR**) **and acoustical parameters**

**Acoustical parameters**	***A***	***R***^**2**^	***D***	***R***^**2**^
*L*_Aeq_	*A* = 1.986*L*_Aeq_-75.46^a^	0.839	*D* = 1.968*L*_Aeq_-79.59^a^	0.816
*SEL*	*A* = 1.986 *SEL*-134.1^a^	0.839	*D* = 1.968 *SEL*-137.7^a^	0.816
*L*_AFmax_	*A* = 1.873*L*_AFmax_-88.63^a^	0.942	*D* = 1.852*L*_AFmax_-92.38^a^	0.913
*L*_ASmax_	*A* = 1.939*L*_ASmax_-84.86^a^	0.884	*D* = 1.917*L*_ASmax_-88.58^a^	0.856
*L*_A10_	*A* = 1.964*L*_A10_-63.40^a^	0.653	*D* = 1.989*L*_A10_-70.19^a^	0.663
*L*_EPN_	*A* = 1.886*L*_EPN_-116.9^b^	0.711	*D* = 1.857*L*_EPN_-119.5^b^	0.683
*L*_N_	*A* = 12.15*L*_N_-740.5^a^	0.830	*D* = 12.34*L*_N_-757.9^a^	0.848
	*A* = 2.218*L*_N_-132.3^b^	0.782	*D* = 2.177*L*_N_-133.9^b^	0.757
*S*	*A* = 171.55*S*-177.1^a^	0.707	*D* = 170.76*S*-181.3^a^	0.695
	*A* = 38.62*S*-16.50^b^	0.751	*D* = 38.52*S*-21.27^b^	0.752
*R*	*A* = -50.91*R* + 103.4^a^	0.341	*D* = -50.06*R* + 97.23^a^	0.327
	*A* = 23.36*R* + 2.172^b^	0.635	*D* = 22.98*R*-1.978^b^	0.618
*F*	*A* = 6009.7 *F* + 26^a^	0.496	*D* = 6183.7 *F* + 19^a^	0.520
	*A* = 2294.1 *F* + 17.87^b^	0.580	*D* = 2324 *F* + 12.48^b^	0.599
*L*_HC_	*A* = *L*_HC_-89.7^a^	0.991	*D* = *L*_HC_-94.8^a^	0.977

^a^over 15 min, ^b^over just 45 s.

## Discussion

To develop the environmental noise limits, the practicality should be also considered. It would be useful to develop a single index which could be applied for both HR and CR noise, namely, with the same limit value when such an index is used. In addition to *L*_Aeq_, relationships between some acoustical parameters and the subjective evaluation results have been analyzed. The acoustical parameters include sound exposure level, *SEL*, which is a indicator on the total noise energy produced by a single noise event; the maximum of the instantaneous fast A-weighted sound pressure level with a duration of 125 ms, *L*_AFmax_; the maximum of the instantaneous slow A-weighted sound pressure level with a duration of 1000 ms, *L*_ASmax_[34,35]; the percentile levels, *L*_A10_, which is exceeded by 10% of measured data during the total measurement time period; in particular [19], the effective perceived noise levels, *L*_EPN_, which can be obtained by analyzing one pass-by sound, based on the calculation method reported in ISO 3891-1978 [36]; and four psychoacoustics parameters including loudness levels (*L*_N_), the sound pressure level of a 1 kHz tone sounding as loud as a noise event; sharpness (*S*), an index describing high-frequency characteristics of a sound sample; roughness (*R*), an indicator on sound qualities associating with amplitude modulations caused by tones above 20 Hz; and fluctuation (*F*), an indicator on sound qualities associating with amplitude modulations caused by tones below 20 Hz [37], both over 15 min including quiet periods and over just 45 s. The results are shown in Table 1.

Generally speaking, the ambient noise is not only from one source. Considering combinations of various noise sources, *L*_Aeq_ is the most commonly used noise descriptor in environmental noise standards and regulations. As shown in Table 2, *L*_Aeq_ is also the most important index for evaluating railway noise. In addition, *L*_AFmax_ and *L*_ASmax_ are normally selected to evaluate the noise with impulsive character. *L*_AFmax_ has been used for emission values for trains [38]. *L*_ASmax_ was selected to evaluate the HR noise by the Environmental Agency of Japan [39] and Taiwan [40].

**Table 2:** **Noise limits** (***L***_**Aeq**_) **of the areas along railways**

**Region**	**Type of area**	**HR noise/dB**		**CR noise/dB**		**Difference between CR and HR/dB**	
		**Daytime**	**Night**	**Daytime**	**Night**	**Daytime**	**Night**
China	-	63	53	70	60	7	7
Belgium	-	60	50	65	60	5	10
France	Hospital, social buildings	60	55	63	58	3	3
	Schools	60	-	63	-	3	-
	Dwellings in pre-existing moderate sound environment zones*	60	55	63	58	3	3
	Other dwellings	65	60	68	63	3	3
	Office buildings in pre-existing moderate sound environment zones	65	-	68	-	3	-
Taiwan	Hospital, school, dwelling	65 (Morning, evening) 70(Day)**	60	73	70	8 (Morning, evening) 3(Day)	10
	Mixed areas, commercial areas, industrial areas	70 (Morning, evening) 75(Day)	65	75	70	5 (Morning, evening) 0(Day)	5

*Moderate sound environment zones are the areas where the environmental noise level existing before the new infrastructure.

**Daytime is divided into morning (5:00-7:00), day (7:00-20:00) and evening (20:00-22:00).

Table 3 shows the *L*_Aeq_ and *L*_p_ over 15 min for different stimuli sampled at different distance from the outboard track. As shown in Table 3, the differences between the total un-weighted SPL and *L*_Aeq_ (*L*_p_-*L*_Aeq_) were 20.0-24.7 dB for HR noise, and 8.3-15.4 dB for CR noise. This suggests that compared with CR noise, the proportion of low frequency sound energy is larger for HR noise, which could result in greater annoyance of HR noise compared with that of CR noise at the same *L*_Aeq_. Through a detailed analysis, it has been proved that the higher low frequency content compared with that of CR could not be due to the noise summation within “no train” period in Figure 2. Generally speaking, as the propagation distance increases, the proportion of low frequency sound energy would also increase. Consequently, *L*_p_-*L*_Aeq_ of CR noise increases monotonically, with increasing distance from the outboard track. For HR, however, due to the influence of elevated track, the *L*_p_-*L*_Aeq_ does not increase monotonically.

**Table 3:** ***L***_**Aeq**_, ***L***_**p **_**and *L***_**Aeq**_-***L***_**p **_**of stimuli sampled at different distance from outboard track of HR and CR**

**Stimuli**		***L***_**Aeq**_	***L***_**p**_	***L***_**p**_**-*L***_**Aeq**_
HR	20 m^a^	75.1	99.8	24.7
	30 m	68.3	88.4	20.1
	50 m	62.7	82.7	20.0
	100 m	58.4	78.6	20.2
	200 m	53.6	76.8	23.2
CR	20 m	76.0	84.6	8.6
	30 m	71.3	81.7	10.4
	50 m	64.4	76.0	11.6
	100 m	58.6	70.9	12.3
	200 m	53.9	67.8	13.9

^a^The distance between noise sampling site and the outboard track.

As a combined acoustical parameter proposed to evaluate the total railway noise, *L*_HC_ = 1.74*L*_AFmax_ + 0.008*L*_AFmax_(*L*_p_-*L*_Aeq_), where *L*_AFmax_ could express the influence of impulsive character, and *L*_p_-*L*_Aeq_ could express the influence of low frequency character, which is very different between HR noise and CR noise as shown in Figure 1 and Figure 2. As a result, it is expected that both HR and CR can be taken into account in the same set of equations. *L*_p_-*L*_Aeq_ will decrease with the interval of train pass-by events increasing, i.e., the influence (*A* or *D*) induced by railway noise will decrease and be lower than that of maximum train pass-by events.

**Figure 4:**
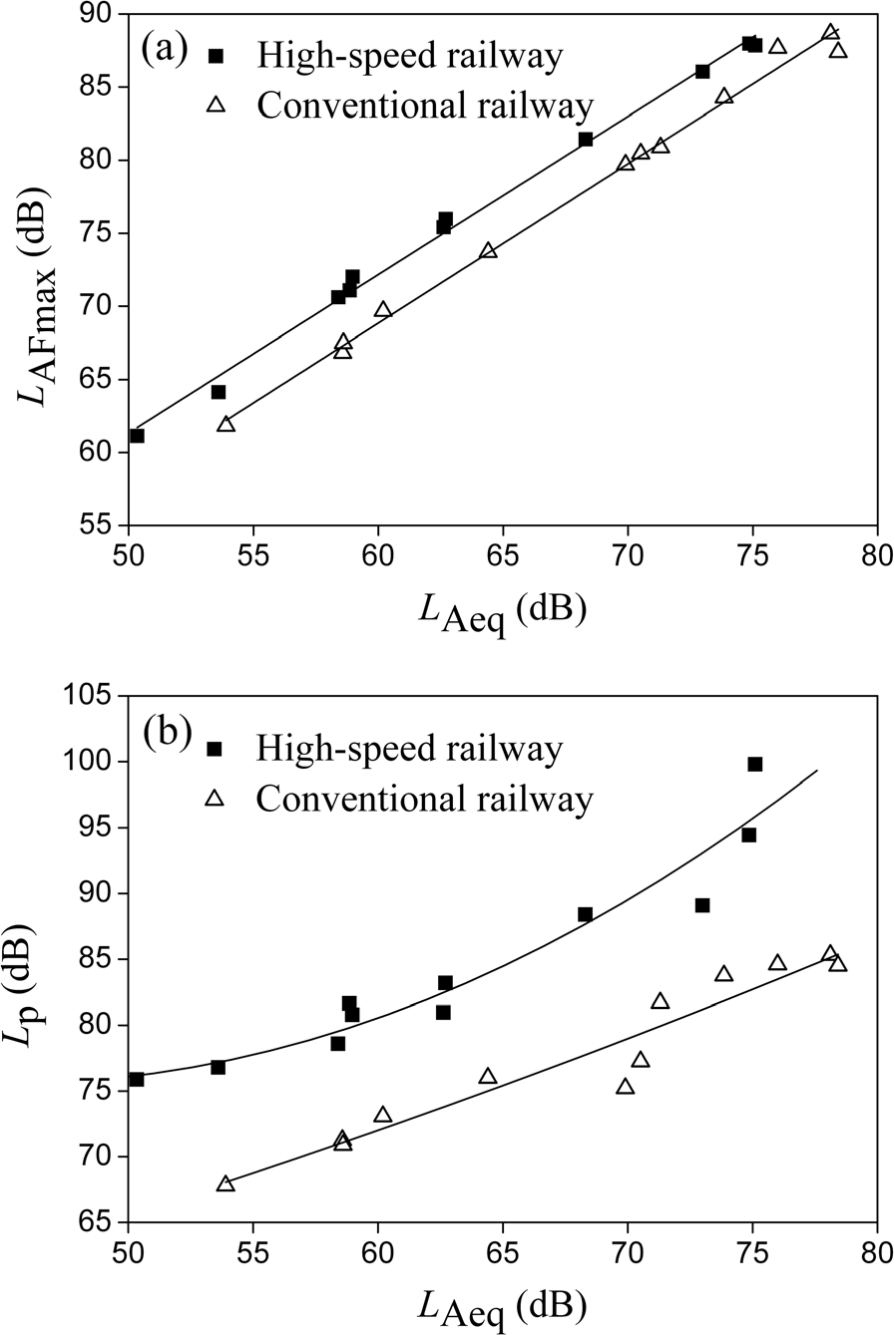
**The relationships between *L***_**AFmax **_**and *L***_**Aeq**_**, and between *L***_**p **_**and *L***_**Aeq**_**. (a)** Between *L*_AFmax_ and *L*_Aeq._ HR: *L*_AFmax_ = 1.08*L*_Aeq_ + 7.2 (*R*^2^ = 0.995); CR: *L*_AFmax_ = 1.09*L*_Aeq_ + 3.4 (*R*^2^ = 0.994); **(b)** Between *L*_P_ and *L*_Aeq._ HR: *L*_p_ = 0.023*L*_Aeq_^2^-2.04*L*_Aeq_ + 121.7 (*R*^2^ = 0.928); CR: $L_{p}=41.443e^{0.0092L_{\mathrm{Aeq}}}$ (*R*^2^ = 0.934).

Compared with regression models of *A* and *D* in *L*_Aeq_ for only HR noise or CR noise (Eq. (4) - Eq. (7)), the combined model of *L*_HC_ for total railway noise (Eq. (8) and - Eq. (9)) has additional advantages as discussed below. In Figure 4 the relationships between acoustical parameters of HR noise and CR noise are given. According to the regression equations between *L*_AFmax_ and *L*_Aeq_, and between *L*_p_ and *L*_Aeq_, as shown in the caption of Figure 4, it can be calculated that when *L*_Aeq_ is 60.0 dB, *L*_AFmax_ and *L*_p_ of HR are 72.0 dB and 82.1 dB; and *L*_AFmax_ and *L*_p_ of CR are 68.8 dB and 71.9 dB, respectively. When *L*_Aeq_ is 70.0 dB, *L*_AFmax_ and *L*_p_ of HR are 82.8 dB and 91.6 dB; *L*_AFmax_ and *L*_p_ of CR are 79.7 dB and 78.9 dB, respectively. Then they are substituted into Eq. (8), and when *L*_Aeq_ is 60.0 dB and 70.0 dB, the difference in *A* between HR noise and CR noise is 11.7 and 14.0, which is not identical. However, according to Figure 3 or Eq. (4) and (5), when *L*_Aeq_ is 60.0 dB and 70.0 dB, the difference in *A* between HR noise and CR noise is 13.1 and 13.0, which is basically identical. Similar results about the difference in *D* between HR noise and CR noise can be derived from Eq. (6), (7) and (9) when *L*_Aeq_ is 60.0 dB and 70.0 dB.

In fact, based on the regression equations between *L*_AFmax_ and *L*_Aeq_, and between *L*_p_ and *L*_Aeq_ given in the caption of Figure 4, Eq. (12) for HR noise and Eq. (13) for CR noise between *L*_HC_ and *L*_Aeq_ can be derived as follows.

(12) $$\begin{array}{l} L_{\mathrm{HC}}=1.74\left(1.08L_{\mathrm{Aeq}}+7.2\right)+0.008\left(1.08L_{\mathrm{Aeq}}+7.2\right)\\ \;\left(0.023{L_{\mathrm{Aeq}}}^{2}-2.04L_{\mathrm{Aeq}}+121.7-L_{\mathrm{Aeq}}\right) \end{array}$$

(13) $$\begin{array}{l} L_{\mathrm{HC}}=1.74\left(1.09L_{\mathrm{Aeq}}+3.4\right)+0.008\left(1.09L_{\mathrm{Aeq}}+3.4\right)\\ \;\left(41.443e^{0.0092L_{\mathrm{Aeq}}}-L_{\mathrm{Aeq}}\right) \end{array}$$

According to Eq. (8), (9), (12) and (13), *L*_HC_ of HR and CR noises with same *A* were calculated and were listed in Table 4. Results indicate that *L*_HC_ of HR and CR noises with same *A* is identical basically.

**Table 4:** ***L***_**Aeq **_**and *L***_**HC **_**of HR and CR noises with same *A***

***A***	***L***_**Aeq**_			***L***_**HC**_		
	**HR**	**CR**	**CR-HR**	**HR**	**CR**	**CR-HR**
25	47.8	54.2	6.4	115.9	115.7	-0.2
28	49.2	55.6	6.4	118.4	118.3	-0.1
31	50.7	57.1	6.4	121.0	121.0	0.0
34	52.1	58.5	6.4	123.6	123.7	0.1
37	53.6	60.0	6.4	126.2	126.3	0.1
40	55.1	61.5	6.4	128.8	128.9	0.1
43	56.5	62.9	6.4	131.5	131.6	0.1
46	58.0	64.4	6.4	134.2	134.2	0.0
49	59.5	65.8	6.3	137.0	136.8	-0.2
52	60.9	67.3	6.4	139.8	139.5	-0.3
55	62.4	68.7	6.3	142.6	142.1	-0.5
58	63.9	70.2	6.3	145.5	144.7	-0.8

These results above suggest that compared with the single model in *L*_Aeq_ for only HR noise or CR noise, the combined model using *L*_HC_ for total railway noise can distinguish the difference of *A* (or *D* ) between HR noise and CR noise with different *L*_Aeq._ Moreover, when *L*_Aeq_ of HR is 63 dB and *L*_Aeq_ of CR is 70 dB, whose annoyance (*A*) is the same, *L*_HC_ is approximately 126; when *L*_Aeq_ of HR is 53 dB and *L*_Aeq_ of CR is 60 dB, *L*_HC_ is approximately 144. In other words, if *A* of HR noise and CR noise with different *L*_Aeq_ is the same, *L*_HC_ will be identical. As a result, compared with *L*_Aeq_, *L*_HC_ would be a better evaluation index of total railway noise. This can also be seen in Table 1, where among various acoustical parameters including *L*_HC_, *L*_Aeq_, *SEL*, *L*_AFmax_, *L*_ASmax_, *L*_A10_, *L*_EPN_, *L*_N_, *S*, *R* and *F*, *A* and *D* have the highest correlation with *L*_HC_.

In many cases, HR and CR noises are coexistent in an actual environment. As the limits of both HR noise and CR noise are different in *L*_Aeq_, each noise has to be distinguished and measured from total railway noise. However, the limit of *L*_HC_ of both HR noise and CR noise is identical, so two types of railway noises can be measured synchronously.

When there are combinations of various noise sources, such as railway and road traffic [41], *L*_Aeq_ might be a more general noise evaluation index. However, a single index, *L*_Aeq_, would not be sufficient to evaluate the influence of railway noise, as discussed above, it might be possible to use multiple indices such as *L*_Aeq_, *L*_p_ and *L*_Amax_ (*L*_AFmax_ or *L*_ASmax_). There might be also a scope of modifying *L*_HC_ for combined noise sources.

It must be noticed that use of young students with perfect hearing in this case study may limit generalizability of findings. More subjective evaluation experiments or epidemiological investigations covering different age and occupation groups may extend the generalizability of findings. In addition, subjects were given a video of the train passing during the noise exposure. It means that subjects could have identified which was high speed train and which was conventional train. This maybe introduced a bias into the subjective reporting of annoyance and activity disturbance.

## Conclusions

Based on binaural recording of high-speed railway and conventional railway noises in a semi-free field, the noise annoyance and activity disturbance induced by maximal train pass-by events in China (six pass-by sounds with a constant interval over 15 min) were investigated through laboratory subjective evaluation. With the same annoyance rating (*A*) or activity disturbance rating (*D*), the A-weighted equivalent sound pressure level (*L*_Aeq_) of CR noise is approximately 7 dB higher than that of HR noise. Linear regression analysis between some acoustical parameters and *A* (or *D*) suggests that the coefficient of determination (R^2^) is higher with the instantaneous fast A-weighted sound pressure level (*L*_AFmax_) than that with *L*_Aeq_. A combined acoustical parameter, *L*_HC_ = 1.74*L*_AFmax_ + 0.008*L*_AFmax_(*L*_p_-*L*_Aeq_), where *L*_p_ is the sound pressure level, was derived consequently, which could better evaluate the total railway noise, including HR and CR noise. More importantly, with a given *L*_HC_, the noise annoyance of HR and CR noise is the same. When there are combinations of various noise sources, there might be a scope of modifying *L*_HC_.

It should be pointed out that this study provides suggestive evidence, rather than a final proof. As a laboratory study involving students in their 20s (not a general population with naturally experienced levels of noise) with potential bias from being able to clearly identify which train was which, the results and conclusions above could have potential limitations. Further study is expected to elucidate conclusions above by additional measurements.

## Abbreviations

CR: Conventional railway; HR: High-speed railway; MLR: Magnetic levitation railway; *A*: Annoyance rating; *D*: Activity disturbance rating; *L*_p_: Sound pressure level; *L*_Aeq_: A-weighted equivalent sound pressure level; *L*_A10_: A-weighted equivalent sound pressure level exceeded for 10% of the time of the measurement duration; *L*_AFmax_: Maximum level with a-weighted frequency response and fast time constant; *L*_ASmax_: Maximum level with a-weighted frequency response and slow time constant; *L*_EPN_: Effective perceived noise level; *L*_N_: Loudness level; *L*_HC_: Noise Level of high-speed railway and conventional railway; *L*_L_: Sound level in left ear of the binaural noise sample; *L*_R_: Sound level in right ear of the binaural noise sample; *L*_S_: Average value of *L*_L_ and *L*_R_ of the binaural noise sample; *F*: Fluctuation level; *R*: Roughness level; *S*: Sharpness level; *SEL*: Sound exposure level; *R*^2^: Coefficient of determination; *D*: Distance between the noise sampling site and the outboard track.

## Competing interests

The authors declare that they have no competing interests.

## Authors’ contributions

All authors have read the final version of the manuscript and have given final approval of this version of the manuscript to be published. GQD conducted the study design, data analysis and completed the final version of the manuscript. QLL planned the experiment, execution of data collection and prepared the first draft of the manuscript. ZGL assisted in the analysis of the study data, participated in the writing of the manuscript. JK participated in the study design, and critically reviewed the manuscript. All authors read and approved the final manuscript.
